# Combination of 2-*tert*-Butyl-1,4-Benzoquinone (TBQ) and ZnO Nanoparticles, a New Strategy To Inhibit Biofilm Formation and Virulence Factors of Chromobacterium violaceum

**DOI:** 10.1128/msphere.00597-22

**Published:** 2023-01-16

**Authors:** Junsheng Liu, Zengyan Chang, Xiaosa Chang, Junjian Li, Ulrich Glebe, Ai-Qun Jia

**Affiliations:** a Key Laboratory of Tropical Biological Resources of Ministry of Education, One Health Institute, Hainan University, Haikou, China; b School of Pharmaceutical Sciences, Hainan University, Haikou, China; c School of Pharmaceutical Sciences, Wuhan University, Wuhan, China; d Institute of Chemistry, University of Potsdam, Potsdam-Golm, Germany; e Fraunhofer Institute for Applied Polymer Research IAP, Potsdam-Golm, Germany; Antimicrobial Development Specialists, LLC

**Keywords:** *Chromobacterium violaceum*, 2-*tert*-butyl-1,4-benzoquinone, ZnO nanoparticles, biofilm, quorum sensing inhibitor

## Abstract

Drug-resistant bacteria have been raising serious social problems. Bacterial biofilms and different virulence factors are the main reasons for persistent infections. As a conditioned pathogen, Chromobacterium violaceum has evolved a vast network of regulatory mechanisms to modify and fine-tune biofilm development, contributing to multidrug resistance. However, there are few therapies to combat drug-resistant bacteria. Quorum sensing (QS) inhibitors (QSIs) are a promising strategy to solve antibiotic resistance. Our previous work suggested that 2-*tert*-butyl-1,4-benzoquinone (TBQ) is a potent QSI. In this study, the combination of zinc oxide nanoparticles (ZnO-NPs) and TBQ (ZnO-TBQ) was investigated for the treatment of Chromobacterium violaceum ATCC 12472 infection. ZnO-NPs attach to cell walls or biofilms, and the local dissolution of ZnO-NPs can lead to increased Zn^2+^ concentrations, which could destroy metal homeostasis, corresponding to disturbances in amino acid metabolism and nucleic acid metabolism. ZnO-NPs significantly improved the efficiency of TBQ in inhibiting the QS-related virulence factors and biofilm formation of C. violaceum ATCC 12472. ZnO-TBQ effectively reduces the expression of genes related to QS, which is conducive to limiting the infectivity of C. violaceum ATCC 12472. Caenorhabditis elegans nematodes treated with ZnO-TBQ presented a significant improvement in the survival rate by 46.7%. Overall, the combination of ZnO-NPs and TBQ offers a new strategy to attenuate virulence factors and biofilm formation synergistically in some drug-resistant bacteria.

**IMPORTANCE** The combination of ZnO-NPs and TBQ (ZnO-TBQ) can compete with the inducer *N*-decanoyl-homoserine lactone (C_10_-HSL) by binding to CviR and downregulate genes related to the CviI/CviR system to interrupt the QS system of C. violaceum ATCC 12472. The downstream genes responding to *cviR* were also downregulated so that virulence factors and biofilm formation were inhibited. Furthermore, ZnO-TBQ presents multiple metabolic disturbances in C. violaceum ATCC 12472, which results in the reduced multidrug resistance and pathogenicity of C. violaceum ATCC 12472. In an *in vivo* assay, C. elegans nematodes treated with ZnO-TBQ presented a significant improvement in the survival rate by 46.7% by limiting the infectivity of C. violaceum ATCC 12472. In addition, ZnO-TBQ inhibited the generation of virulence factors and biofilm formation 2-fold compared to either ZnO-NPs or TBQ alone. The combination of ZnO-NPs with TBQ offers a potent synergistic strategy to reduce multidrug resistance and pathogenicity.

## INTRODUCTION

In recent years, the resistance of pathogens to antimicrobial agents has been alarming. The lack of new and effective antimicrobials has resulted in a global public health threat according to the WHO (1). Bacteria have a >4-times-higher survival rate in old biofilms than in young biofilms (2). By forming a biofilm, bacteria protect themselves against antibiotics. With the maturation of a biofilm, its antibiotic tolerance sharply increases (3). This is a social behavior in bacterial communities in response to environmental changes. This adaptive behavior is regulated by quorum sensing (QS), a density-dependent system of cell-to-cell communication that relies on the production and release of small-molecule signals, termed autoinducers, into the extracellular environment (4). One of the main groups of autoinducers identified as QS signals in Gram-negative bacteria is the *N*-acyl-l-homoserine lactones (AHLs) (5).

The formation of biofilms, accompanied by higher levels of antimicrobial resistance, is a serious threat to public as well as domestic health. Thus, inhibiting biofilm formation is an effective way to combat antimicrobial resistance (6). Previous studies have unveiled that the formation of a biofilm and the secretion of virulence factors are directly or indirectly related to QS (7). Consequently, identifying potent quorum sensing inhibitors (QSIs) that inhibit the formation of biofilms and the secretion of virulence factors could be an efficient mean to tackle the challenge of drug-resistant bacteria.

Current approaches to the eradication of biofilms use nonspecific broad-spectrum antibiotics. The need for new antimicrobial therapies indicates that alternative strategies must be developed urgently (8). In this regard, nanoparticles (NPs) are currently attracting global attention. Nanoparticles have proven effective in treating infectious diseases *in vitro* and *in vivo* and could even treat infections caused by antibiotic-resistant bacterial strains (9). Nanoparticles could become an indispensable viable therapeutic option for the treatment of drug-resistant infections. Of all of the nanoparticles, metal oxide nanoparticles appear to offer the most promise and have attracted tremendous interest from many researchers (1). ZnO nanoparticles (ZnO-NPs) have been widely reported for their antibacterial and antibiofilm activities against a broad spectrum of microbes such as Staphylococcus aureus, Enterococcus faecalis, Escherichia coli, Klebsiella pneumoniae, and Pseudomonas aeruginosa, with low toxicity to human cells (10). Antibacterial activity was shown to depend on the size of the nanoparticles (11). Nanoparticles of <50 nm demonstrated a higher level of activity than larger particles (such as 250 or 350 nm) (12). However, the use of NPs together with QSIs has rarely been studied for the inhibition of biofilm formation. The combination of QSIs and nanomaterials to curb quorum sensing is a recently developed effective strategy that could help to tackle the challenge of antibiotic resistance (13).

Chromobacterium violaceum is an example of an opportunistic pathogen (14). It has evolved a vast network of regulatory mechanisms to modify and fine-tune biofilm development. C. violaceum frequently causes sepsis and distant metastasis. Together with its multidrug resistance, frequent relapses, and high mortality rate, C. violaceum poses a social concern. Moreover, this bacterium is completely resistant to some antibiotics (15).

Our previous studies indicated that 2-*tert*-butyl-1,4-benzoquinone (TBQ) acts as a QSI against C. violaceum ATCC 12472 (16). Here, the aim of this study is to investigate if the combination of ZnO-NPs with TBQ (ZnO-TBQ) results in a synergistic effect on the inhibition of virulence factors and biofilm formation. We found that ZnO-NPs inhibited biofilm formation by C. violaceum ATCC 12472. At a concentration of 25 μg/mL, ZnO-NPs improved the activity of TBQ. Moreover, ZnO-NPs and TBQ together inhibited the generation of virulence factors and biofilm formation 2-fold compared to either ZnO-NPs or TBQ alone. The treatment of Caenorhabditis elegans nematodes with ZnO-TBQ presented a significant improvement in the survival rate by 46.7% by limiting the infectivity of C. violaceum ATCC 12472. Therefore, TBQ and ZnO-NPs have been found to enhance their antibiofilm properties synergistically and to inhibit virulence factors.

## RESULTS

ZnO-NPs of a 40-nm diameter were used for all experiments. To investigate their possible synergistic effects, ZnO-NPs and TBQ were mixed at a ratio of 1:1 (mass ratio). Biofilms of C. violaceum ATCC 12472 were grown in the presence of ZnO-NPs, TBQ, ZnO-TBQ (25 μg/mL each), or dimethyl sulfoxide (DMSO) as a negative control. The MICs of ZnO-NPs, TBQ, and ZnO-TBQ for C. violaceum ATCC 12472 were determined, and the biofilms were investigated by scanning electron microscopy (SEM) and confocal laser scanning microscopy (CLSM) after treatment. Real-time quantitative PCR (RT-qPCR) was used to analyze changes in bacterial quorum sensing (QS) gene expression upon treatment with ZnO-NPs, TBQ, or ZnO-TBQ. A C. elegans survival assay was used to assess the survival rate upon infection with C. violaceum ATCC 12472.

### MIC and growth profile.

**FIG 1:**
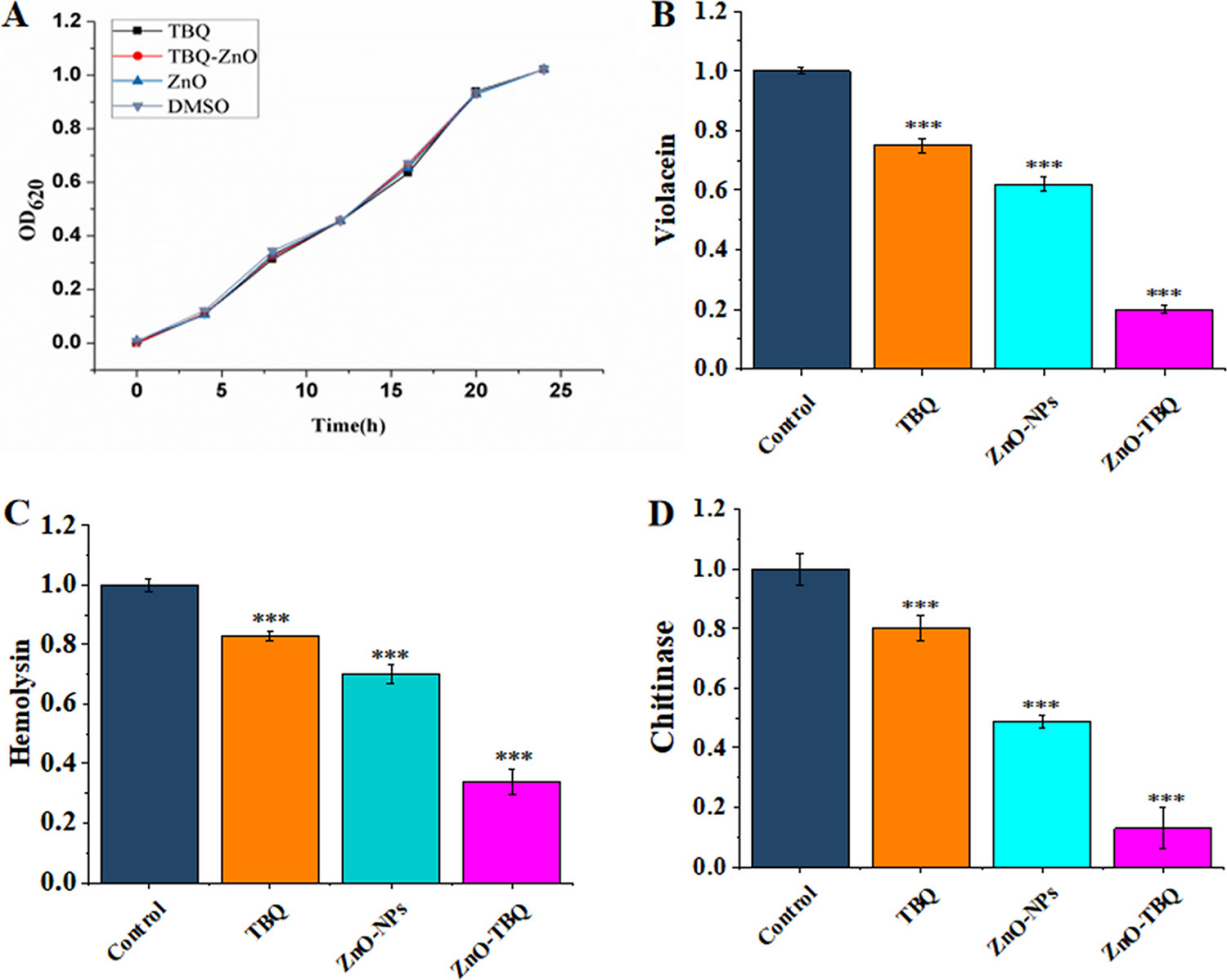
Growth profiles of C. violaceum ATCC 12472 during treatment with TBQ, ZnO-NPs, and ZnO-TBQ (25 μg/mL each) (A) and inhibitory effects of TBQ, ZnO-NPs, and ZnO-TBQ on the production of the virulence factors violacein (B), hemolysin (C), and chitinase (D). DMSO served as a negative control. The error bars represent the standard deviations from three measurements. Statistical differences were determined by ANOVA followed by a Tukey-Kramer test. **, *P < *0.01 versus the DMSO control; ***, *P < *0.001 versus the DMSO control.

It is noteworthy that C. violaceum, as an opportunistic human pathogen, presents multidrug resistance characteristics and the possibility of relapse (17). Due to their high levels of β-lactamase, most strains of C. violaceum are resistant to β-lactam antibiotics, for example, cephalosporin and penicillins. In addition, this bacterium has shown resistance to rifampin and vancomycin, which renders its treatment difficult (18). To investigate the susceptibility of C. violaceum ATCC 12472 to ZnO-NPs, TBQ, and ZnO-TBQ, the corresponding MICs were determined using a 2-fold gradient dilution method. As shown in Table S1 in the supplemental material, the MICs of TBQ, ZnO-NPs, and ZnO-TBQ for C. violaceum ATCC 12472 were 100 μg/mL, 100 μg/mL, and 50 μg/mL (Table S1), respectively. The growth profile of C. violaceum ATCC 12472 (Fig. 1A) showed that at 25 μg/mL, TBQ, ZnO-NP, and ZnO-TBQ treatments have no effect on growth.

10.1128/msphere.00597-22.7 TABLE S1 MICs values of ZnO-NPs, TBQ, and ZnO-TBQ. Download Table S1, PDF file, 0.05 MB. Copyright © 2023 Liu et al. 2023 Liu et al. https://creativecommons.org/licenses/by/4.0/ This content is distributed under the terms of the Creative Commons Attribution 4.0 International license.

### Inhibitory effect on virulence factors.

Both the virulence factors and biofilms of C. violaceum are regulated by QS and render this bacterium highly resistant to multiple antibiotics. Quorum sensing is a density-dependent communication system widely found in microbes. Thus, it is a vital strategy to disrupt or inhibit the QS-related virulence factors of C. violaceum without causing pressure to inhibit bacterial growth during the treatment of chronic infection, which could reduce its resistance to antibiotics. C. violaceum characteristically produces violacein, a water-insoluble purple pigment with antibacterial activity (19) whose synthesis is controlled by QS (20). As shown in Fig. 1B, a significant decrease in violacein was observed after treatment with TBQ, ZnO-NPs, and ZnO-TBQ (25 μg/mL each), and violacein production was inhibited by 25%, 38%, and 80%, respectively. Compared with TBQ and ZnO-NPs, the inhibition rate of ZnO-TBQ was increased by 55% and 42%, respectively. Hemolysin, another main virulence factor, which can lyse red blood cells and is related to the pathogenicity of many pathogenic bacteria (21), was reduced by 17%, 30%, and 66% (Fig. 1C) after exposure to TBQ, ZnO-NPs, and ZnO-TBQ (25 μg/mL each), respectively. Compared with TBQ and ZnO-NPs, the inhibition rate of ZnO-TBQ increased by 49% and 30%, respectively. In addition, chitinase was significantly decreased by 20%, 51%, and 87% (Fig. 1D) following the addition of ZnO-NPs, TBQ, and ZnO-TBQ, respectively. Compared with TBQ and ZnO-NPs, the inhibition rate of ZnO-TBQ increased by 67% and 36%, respectively. These results suggest that after exposure to TBQ, ZnO-NPs, and ZnO-TBQ (each at 25 μg/mL), the QS-related virulence factors of C. violaceum ATCC 12472 were significantly decreased without causing pressure to inhibit growth. Remarkably, the ZnO-TBQ combination leads to a dramatic decrease in the virulence factors of C. violaceum ATCC 12472 compared with treatment with ZnO-NPs or TBQ alone.

### Inhibitory effect on biofilm formation.

**FIG 2:**
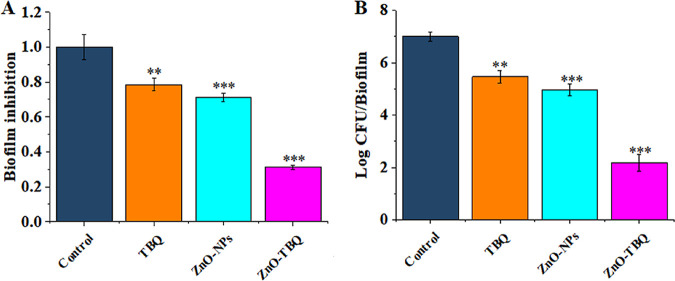
Inhibitory effects of TBQ, ZnO-NPs, and ZnO-TBQ (25 μg/mL each) on biofilms and cell viability. Quantification of the biofilms was performed by a crystal violet staining assay (A), and cell viability was investigated by a spreading plate assay with LB agar medium (B). DMSO served as a negative control. The error bars represent the standard deviations from three measurements. Statistical differences were determined by ANOVA followed by a Tukey-Kramer test. **, *P < *0.01 versus the DMSO control; ***, *P < *0.001 versus the DMSO control.

Bacteria change phenotypically in response to their environment. Free-swimming cells transition to biofilm communities that promote cellular cooperativity and resistance to stressors and antibiotics. Biofilms are the first barrier to antimicrobial drugs entering cells and are one of the main causes of pathogenetic microbial drug resistance (22). After a C. violaceum biofilm forms, drug resistance is unavoidable (23). Thus, the inhibitory effects of TBQ, ZnO-NPs, and ZnO-TBQ on biofilm formation were investigated by a crystal violet assay. At a concentration of 25 μg/mL, the biofilms were markedly reduced by 22%, 29%, and 69% (Fig. 2A), respectively. Compared with TBQ and ZnO-NPs, the inhibition rate of ZnO-TBQ increased by 47% and 40%, respectively. The cell viability in the biofilms was also determined following exposure to ZnO-NPs, TBQ, and ZnO-TBQ. Compared with the control, TBQ, ZnO-NPs, and ZnO-TBQ resulted in fewer viable cells (Fig. 2B). These results revealed that ZnO-TBQ leads to the much more pronounced inhibition of biofilm formation and cell viability of C. violaceum ATCC 12472 than with TBQ and ZnO-NPs. In addition, to evaluate whether the effects on the virulence factors and biofilms of C. violaceum ATCC 12472 at a lower concentration arise from Zn^2+^ ions, ZnO-NPs, or other types of nanoparticles, we investigated the inhibitory effects of Zn(NO_3_)_2_ (1.6 μg/mL [the solubility of ZnO]), Zn(NO_3_)_2_ (1.6 μg/mL)-TBQ (25 μg/mL), ZnO-NPs, ZnO-TBQ, TiO_2_-NPs, and TiO_2_-TBQ (each 2at 5 μg/mL) on violacein, biofilm, and cell viability. As shown in Fig. S1, a significant decrease in violacein was observed after exposure to Zn(NO_3_)_2_-TBQ, ZnO-NPs, ZnO-TBQ, and TiO_2_-TBQ but not after exposure to Zn(NO_3_)_2_ alone and TiO_2_-NPs. This indicates that ZnO-NPs have a greater inhibitory effect than Zn(NO_3_)_2_ alone and TiO_2_-NPs. Compared with Zn(NO_3_)_2_, ZnO-NPs present remarkable inhibitory effects on violacein and cell viability (Fig. S2B), which suggests that the concentration of dissolved Zn^2+^ ions in ZnO-dispersed medium was too low to reduce violacein and bacterial viability in the biofilm (24). Compared with TiO_2_-TBQ, ZnO-TBQ possesses a conspicuous inhibitory effect on violacein and biofilm formation (Fig. S2A), indicating that the inhibitory effect depends on both TBQ and the characteristics of the nanoparticles rather than the nanoparticulate form as such.

10.1128/msphere.00597-22.1 FIG S1 Inhibitory effects of Zn(NO_3_)_2_ (1.6 μg/mL), Zn(NO_3_)_2_ (1.6 μg/mL)-TBQ (25 μg/mL), ZnO-NPs, ZnO-TBQ, TiO_2_-NPs, and TiO_2_-TBQ (25 μg/mL each) on violacein, quantitatively (A) and qualitatively (B). DMSO served as a negative control. The error bars represent the standard deviations from three measurements. Statistical differences were determined by ANOVA followed by a Tukey-Kramer test. **, *P < *0.01 versus the DMSO control; ***, *P < *0.001 versus the DMSO control. Download FIG S1, TIF file, 0.6 MB. Copyright © 2023 Liu et al. 2023 Liu et al. https://creativecommons.org/licenses/by/4.0/ This content is distributed under the terms of the Creative Commons Attribution 4.0 International license.

10.1128/msphere.00597-22.2 FIG S2 Inhibitory effects of Zn(NO_3_)_2_ (1.6 μg/mL), Zn(NO_3_)_2_ (1.6 μg/mL)-TBQ (25 μg/mL), ZnO-NPs, ZnO-TBQ, TiO_2_-NPs, and TiO_2_-TBQ (25 μg/mL each) on biofilms and cell viability. Quantification of the biofilms was performed by a crystal violet staining assay (A), and cell viability was investigated by a spreading plate assay with LB agar medium (B). DMSO served as a negative control. The error bars represent the standard deviations from three measurements. Statistical differences were determined by ANOVA followed by a Tukey-Kramer test. **, *P < *0.01 versus the DMSO control; ***, *P < *0.001 versus the DMSO control. Download FIG S2, TIF file, 0.7 MB. Copyright © 2023 Liu et al. 2023 Liu et al. https://creativecommons.org/licenses/by/4.0/ This content is distributed under the terms of the Creative Commons Attribution 4.0 International license.

### Microscopy analysis of biofilm formation.

**FIG 3:**
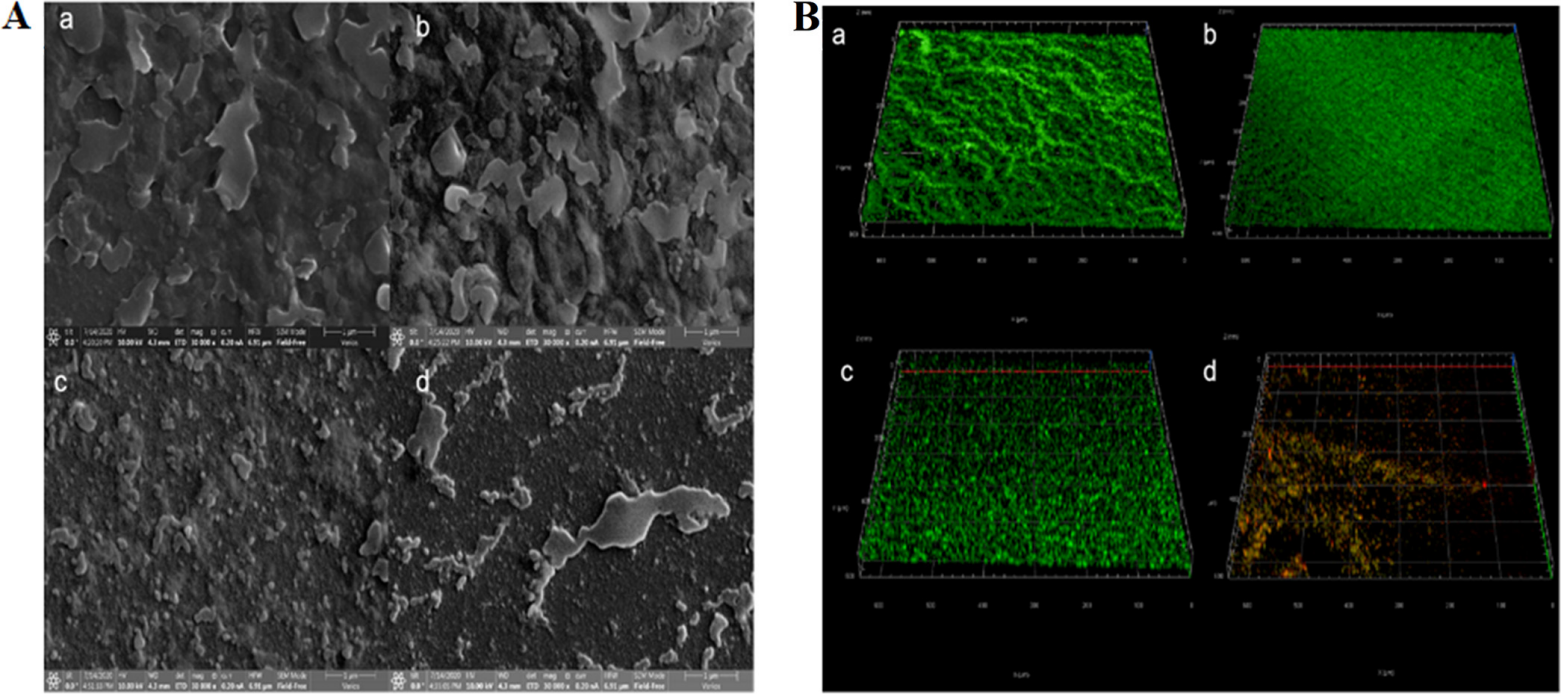
SEM (A) and CLSM (B) images of C. violaceum ATCC 12472 biofilms treated with DMSO (a), TBQ (b), ZnO-NPs (c), and ZnO-TBQ (d) (25 μg/mL each). In the CLSM images, green fluorescence represents viable cells, and red fluorescence represents dead cells.

Scanning electron microscopy (SEM) images were taken from the biofilms before and after treatment (Fig. 3A). The control group (Fig. 3Aa) formed a thick, large, and heterogeneous biofilm. In contrast, the architecture of the biofilms had been destroyed and became thin, scattered, and fragmentized when treated with TBQ (Fig. 3Ab), ZnO-NPs (Fig. 3Ac), and ZnO-TBQ (Fig. 3Ad). After treatment, the internal cells in the biofilms had been exposed to the external environment. Compared with TBQ and ZnO-NPs, ZnO-TBQ exhibited greatly increased activity. These results are in line with the above-described observations on biofilm formation (Fig. 2A). Images obtained by CLSM (Fig. 3B) were similar to the SEM results and support this conclusion. Compared with the control group (Fig. 3Ba), the biofilm architecture appeared sparse and loose, without wrinkles, when treated with TBQ (Fig. 3Bb), ZnO-NPs (Fig. 3Bc), and ZnO-TBQ (Fig. 3Bd). The biofilm thickness was reduced. ZnO-NPs enhanced TBQ’s inhibition of the biofilm formation of C. violaceum ATCC 12472, and the dominant red fluorescence in the combination group indicated that many bacteria had died. Thus, these observations demonstrate the potential of TBQ, ZnO-NPs, and ZnO-TBQ to disrupt biofilm maturation, leading to the formation of weaker biofilms. Therefore, it can be proposed that the use of nanoparticles as adjunctive agents could increase the antimicrobial susceptibility of biofilms and further enhance the efficacy of TBQ.

### QS and virulence-related gene expressions.

**FIG 4:**
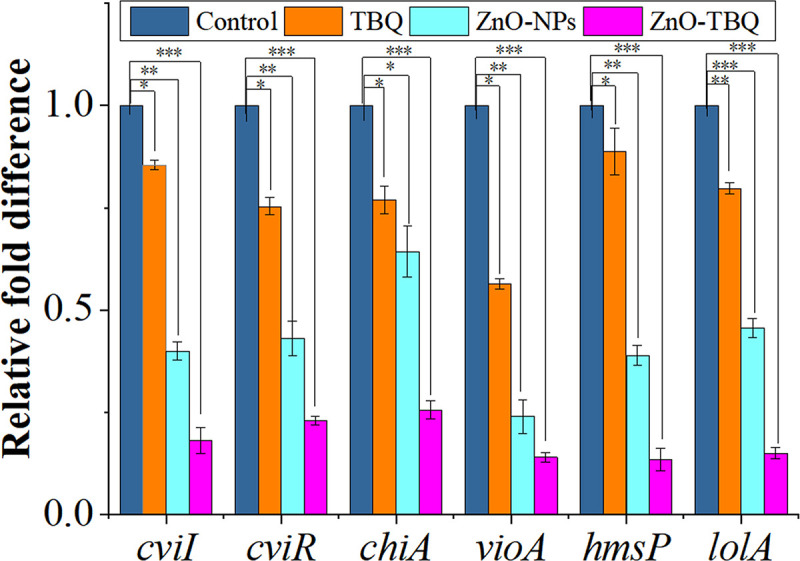
Expression of QS-related genes in C. violaceum ATCC 12472 treated with DMSO, TBQ, ZnO-NPs, and ZnO-TBQ (25 μg/mL each). *, *P < *0.05 versus the control; **, *P < *0.01 versus the control; ***, *P < *0.001 versus the control.

To better understand and assess the enhanced effect of the QSI TBQ in combination with ZnO-NPs, we performed RT-qPCR to determine the mechanisms of the inhibition of the biofilms and virulence factors of C. violaceum ATCC 12472. As shown in Fig. 4, the administration of TBQ, ZnO-NPs, and ZnO-TBQ significantly downregulated the expression of QS-related genes. The LuxI family AHL synthase gene *cviI* was repressed by 15%, 60%, and 82%, respectively. The LuxR family transcriptional regulator gene *cviR* was downregulated by 25%, 57%, and 77%, respectively. Genes encoding various virulence factors, namely, the chitinase synthesis gene *chiA* (by 23%, 36%, and 74%, respectively) and the violacein-related gene *vioA* (by 44%, 76%, and 86%, respectively), were downregulated. The biofilm formation regulator gene *hmsP* was repressed by 11%, 61%, and 87%, respectively, and the outer membrane lipoprotein chaperone gene *lolA* was downregulated by 20%, 54%, and 85%, respectively. The expression of QS-related genes was significantly more downregulated after exposure to ZnO-TBQ than after exposure to TBQ and ZnO-NPs.

### Molecular docking analysis.

**FIG 5:**
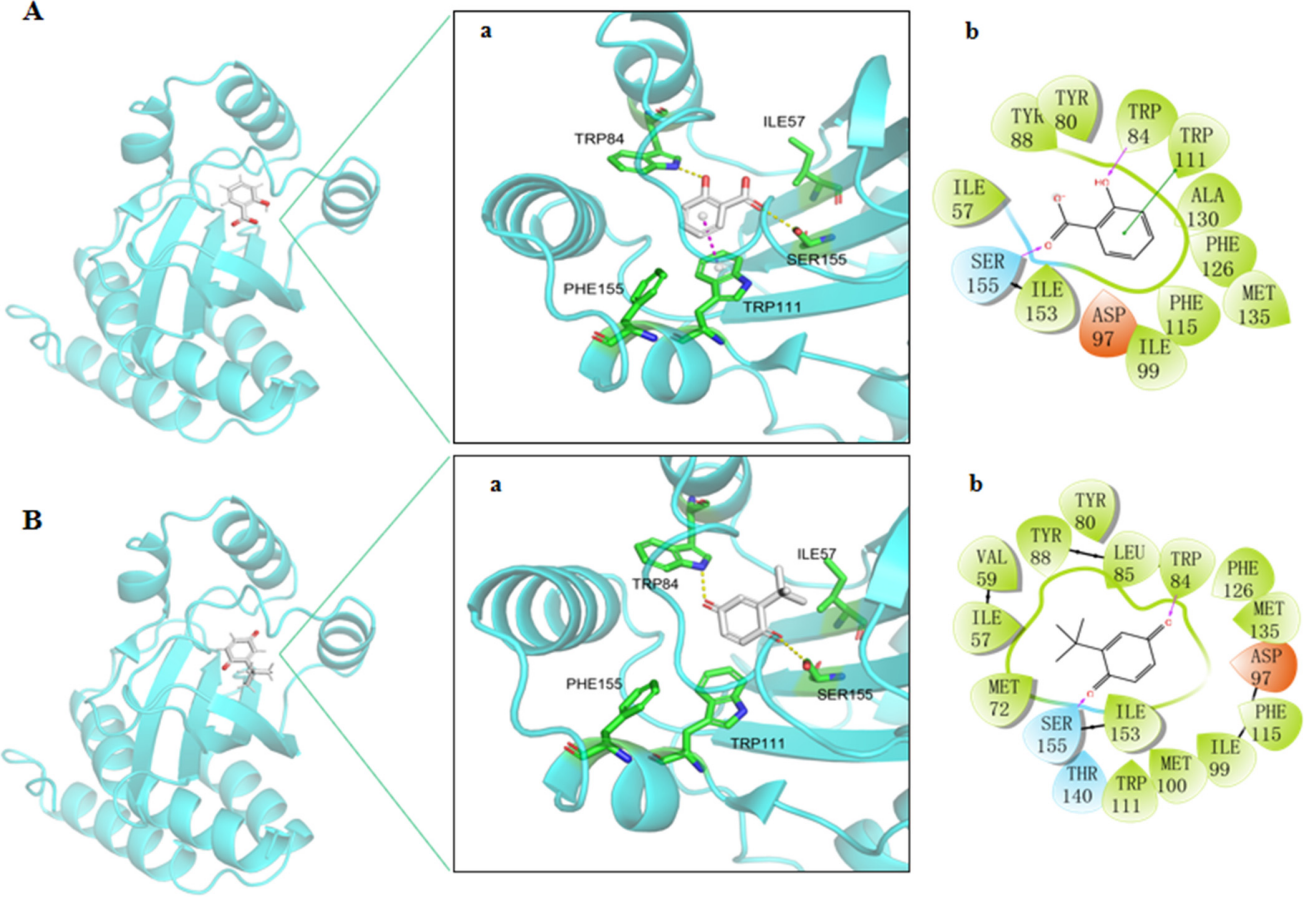
Analysis of the binding modes of salicylic acid and TBQ. (A) Binding mode of salicylic acid in the CviR domain. (B) Binding mode of TBQ in the CviR domain. The three-dimensional (3D) structure and enlarged binding domain (a) and the 2D structure (b) are shown; CviR (PDB accession number 3QP8) is shown in cyan. The carbon atoms of salicylic acid and TBQ are shown in white, and the oxygen atoms are in red. Hydrogen bonds and π-π stacking are indicated with dashed lines.

In view of the above-described observations on the excellent potency of TBQ *in vitro*, it was further assessed by molecular docking analysis to investigate its modes of binding to CviR. With the help of molecular docking analysis, we can figure out if TBQ interferes with the binding of CviR to the CviR receptor. Consequently, the molecular mechanism of the inhibition of QS could be determined. It is worth noting that salicylic acid has been reported to be an effective QSI to interact with proteins that play a role in quorum sensing, reactive oxygen species accumulation, motility, extracellular polymeric matrix components, transport, and metabolism (25). Therefore, it was used as a positive control for comparison. As shown in Fig. 5B, two carbonyl groups of TBQ form hydrogen bonds with the hydroxyl group of Ser155 and the −NH group of Trp84. A carbonyl group of salicylic acid forms the same hydrogen bond with the hydroxyl group of Ser155 as TBQ. The hydroxyl group of salicylic acid forms a hydrogen bond with the −NH group of Trp84. In addition, the benzene ring of salicylic acid forms a π-π stacking interaction with Trp111. Thus, the carbonyl groups of both TBQ and salicylic acid participate in binding to CviR (Fig. 5). More notably, comparing the binding patterns of TBQ with those of salicylic acid, the *tert*-butyl group of TBQ strengthened the extra hydrophobic interaction with Le85, Val59, and Met72 in the active pocket.

### Metabolomics analysis.

Metabolomics analysis was applied to investigate the metabolites related to membrane composition, virulence factor synthesis, and functional protein synthesis. Principal-component analysis (PCA) scatterplots showed a distinct segregation of samples between the control group and the experimental groups (Fig. S3). Projections to latent structures discriminant analysis (PLS-DA) was conducted as a supervised method to identify ion peaks that could be used to differentiate the metabolite profiles of the control group and the experimental groups (Fig. S4). Orthogonal PLS-DA (OPLS-DA) served as a supervised method for pattern recognition, and all comparisons were valid and robust, implying that the differences between the control group and the experimental groups played an important role in differentiating the groups (Fig. S5). A volcano map is essentially used to show scatterplots of the differences (Fig. S6).

10.1128/msphere.00597-22.3 FIG S3 PCA of metabolites of C. violaceum ATCC 12472 treated with TBQ (A), ZnO-NPs (B), and ZnO-TBQ (C). PCA scatterplots show the distinct segregation of samples between the control group and the experimental groups, which suggests that the results are viable. Download FIG S3, TIF file, 1.8 MB. Copyright © 2023 Liu et al. 2023 Liu et al. https://creativecommons.org/licenses/by/4.0/ This content is distributed under the terms of the Creative Commons Attribution 4.0 International license.

10.1128/msphere.00597-22.4 FIG S4 PLS-DA of metabolites of C. violaceum ATCC 12472 treated with TBQ (A), ZnO-NPs (B), and ZnO-TBQ (C). PLS-DA was conducted as a supervised method to identify ion peaks that could be used to differentiate the metabolite profiles of the control group and the experimental groups. To prevent model overfitting during modeling, the model was further tested by a permutation test to ensure model validity. By randomly changing the order of classification variable *Y*, the corresponding PLS-DA model is established multiple times (*n* = 200) to obtain the *R*^2^ and *Q*^2^ values of the random model. The evaluation standard of the permutation test is the intercept between the *Q*^2^ regression line and the *y* axis. From the figure above, the intercept between the *Q*^2^ regression line and the *y* axis is <0.05, and *Q*_2_ is <0, indicating that the PLS-DA model can effectively distinguish samples and can be used for subsequent differential component analysis. Download FIG S4, TIF file, 0.2 MB. Copyright © 2023 Liu et al. 2023 Liu et al. https://creativecommons.org/licenses/by/4.0/ This content is distributed under the terms of the Creative Commons Attribution 4.0 International license.

10.1128/msphere.00597-22.5 FIG S5 OPLS-DA of metabolites of C. violaceum ATCC 12472 treated with TBQ (A), ZnO-NPs (B), and ZnO-TBQ (C). According to OPLS-DA, the *P* value of differential metabolite screening should be ≤0.05, and the VIP value should be >1. Meanwhile, a fold change of ≥1.0 is expressed as upregulated metabolites, and a fold change of <1.0 is expressed as downregulated metabolites. Download FIG S5, TIF file, 0.2 MB. Copyright © 2023 Liu et al. 2023 Liu et al. https://creativecommons.org/licenses/by/4.0/ This content is distributed under the terms of the Creative Commons Attribution 4.0 International license.

10.1128/msphere.00597-22.6 FIG S6 Volcano images of metabolites of C. violaceum ATCC 12472 treated with TBQ (A), ZnO-NPs (B), and ZnO-TBQ (C). The volcano map is essentially used to show the scatterplots of the differences. Spots in blue show downregulated metabolites. Spots in gray show unchanged metabolites. Spots in red show upregulated metabolites. Download FIG S6, TIF file, 0.3 MB. Copyright © 2023 Liu et al. 2023 Liu et al. https://creativecommons.org/licenses/by/4.0/ This content is distributed under the terms of the Creative Commons Attribution 4.0 International license.

**FIG 6:**
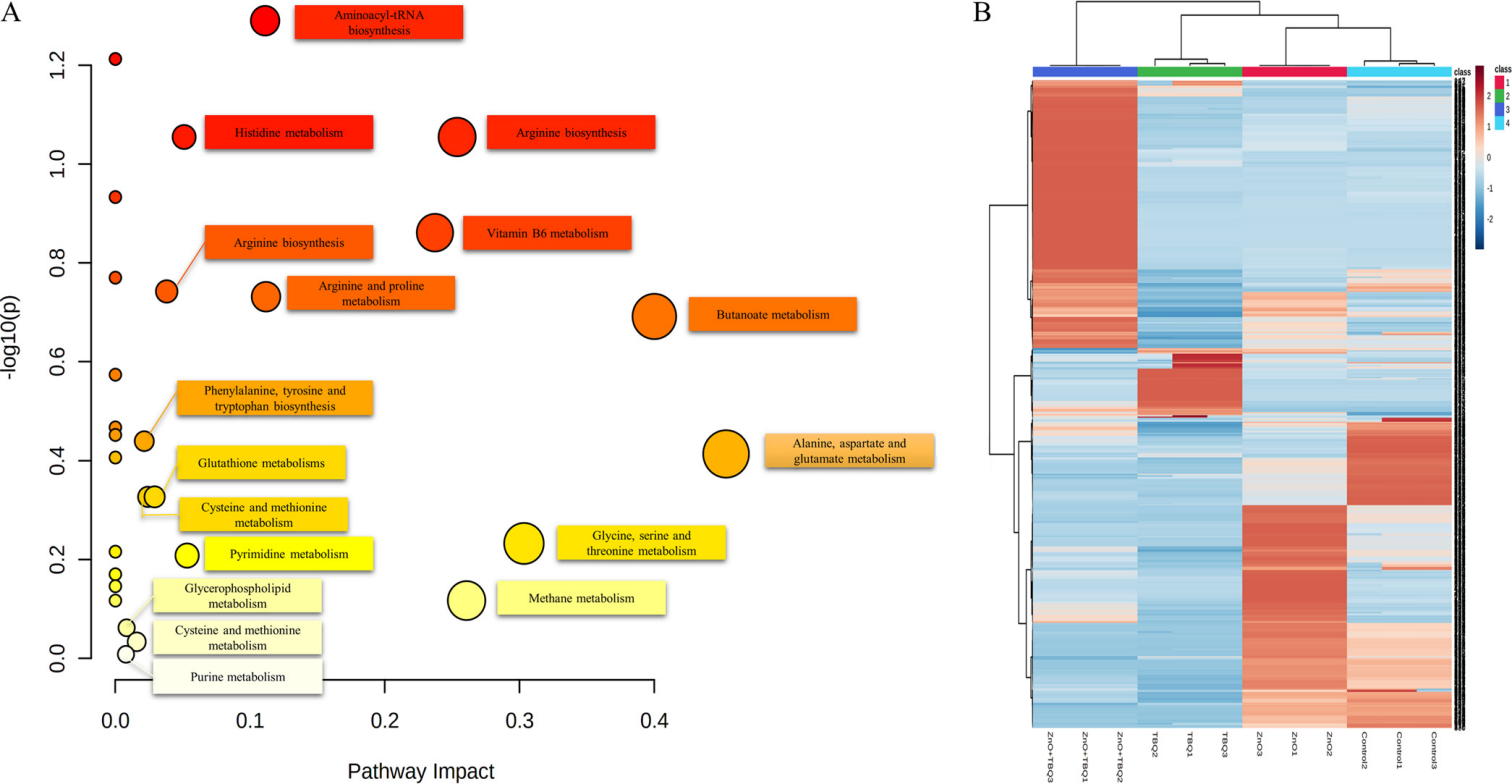
KEGG pathways of the differential metabolites in the control group and the experimental groups (A) and hierarchical clustering analysis heat map of C. violaceum ATCC 12472 (B).

Ultrahigh-performance liquid chromatography–Q-Exactive mass spectrometry (UHPLC-QE-MS) analysis showed dramatic variability after treatment with TBQ, ZnO-NPs, and ZnO-TBQ. According to the criteria of a variable importance in projection (VIP) score of >1 and a *P* value of <0.05, 80 differential metabolites were identified. Among them, 33 differential metabolites were identical in the TBQ, ZnO-NP, and ZnO-TBQ groups. Compared to the control group, 25 metabolites were downregulated: l-glutamic acid, 2-piperidinone, 2-phenylethyl-β-d-glucopyranoside, choline, l-threonine, α-methylstyrene, 7-aminomethyl-7-carbaguanine, 6-methyltetrahydropterin, d-aspartic acid, 5,6-dihydroxyindole, 2-(methylamino)benzoic acid, asymmetric dimethylarginine, 3-amino-butyric acid, l-serine, triethanolamine, xanthine, l-valine, proline, *N*-acetyl-l-leucine, l-phenylalanine, pyridoxine, ornithine, phytosphingosine, l-tyrosine, uracil, l-methionine, and 3-methylhistidine. Eight metabolites were upregulated: isopropyl-d-glucoside, l-norleucine, 1-pyrrololine-2-carboxylic acid, 2′-*O*-methyladenosine, perlolyrine, xanthurenic acid, l-targinine, and *N*-acetyl-l-glutamate-5-semialdehyde. KEGG enrichment analysis (Fig. 6) was performed to gain a further understanding of the metabolic disturbances between the exposures of the control group and the experimental groups to TBQ, ZnO-NPs, and ZnO-TBQ. The impacted pathways mainly interfered in amino acid metabolism, organic acid metabolism, methane metabolism, vitamin B_6_ metabolism, nucleic acid metabolism, sphingolipid metabolism, glutathione metabolism, and glycerophospholipid metabolism.

### *In vivo* Caenorhabditis elegans survival assay.

**FIG 7:**
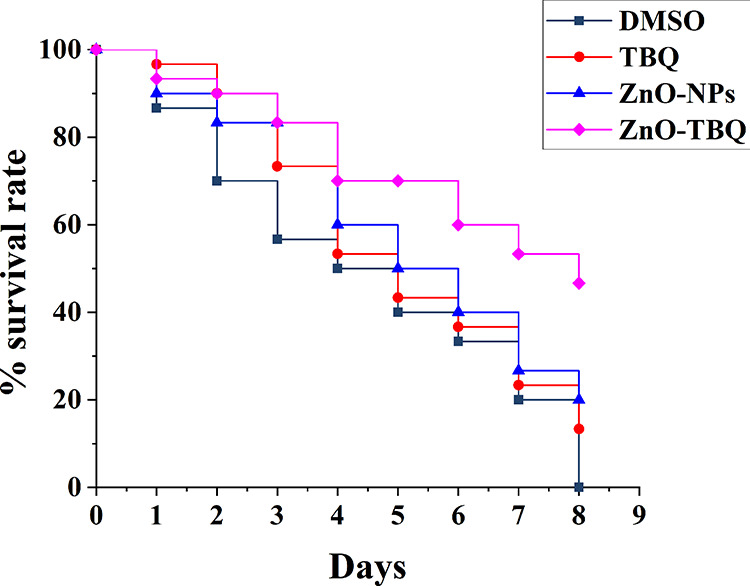
Effects of DMSO (black), TBQ (red), ZnO-NPs (blue), and ZnO-TBQ (purple) (25 μg/mL each) on the rate of survival of C. elegans nematodes infected with C. violaceum ATCC 12472.

C. elegans is a common animal model to assess the toxicity of drugs and the survival rate upon infection with bacteria, which can be combined with treatment with drugs (26). We used C. violaceum ATCC 12472 to infect the model organism C. elegans. When grown in the presence of TBQ, ZnO-NPs, and ZnO-TBQ, the survival rates after 8 days of incubation were 13.3%, 20%, and 46.7%, respectively (Fig. 7). On the other hand, no survival of C. elegans could be detected when the nematodes were grown in the absence of the test compounds. The improvement in the survival rate of the infected C. elegans nematodes upon treatment with the test compounds indicated the attenuation of the virulence of C. violaceum ATCC 12472.

## DISCUSSION

It is known that the inhibition of QS can affect bacterial behaviors in response to environmental changes (27). Quorum sensing inhibitors have been widely researched in recent years to interrupt the QS system. However, the efficiency of the inhibition of the signal molecule is not strong enough, and the rate of inhibition of biofilms is low. Hence, therapies that rely only on current QSIs are not imaginable. Various nanoparticles have been included in bacterial QS research to evaluate the impact of nanoparticles on bacterial infections (28). In a previous report on ZnO-NPs, biofilm formation by ciprofloxacin-resistant Escherichia coli and Proteus mirabilis was inhibited at 100 μg/mL (29). ZnO-NPs also have an impact on the inhibition of biofilms of Pseudomonas aeruginosa PAO1, corresponding to the enhanced susceptibility of PAO1 to antibiotics (30). Considering the excellent antibacterial activity of ZnO-NPs, an obvious hypothesis is that the combination of ZnO-NPs and QSIs could result in synergistic effects on the inhibition of virulence factors and biofilm formation. Our previous study showed that TBQ possesses a QS-inhibitory effect on the pathogenic bacterium C. violaceum ATCC 12472. In addition, the combination of TBQ (50 μg/mL) and ciprofloxacin (sub-MIC, 0.2 μg/mL) synergistically inhibited biofilm formation by 73.27% (16). Therefore, the effects of ZnO-NPs (25 μg/mL) on the production of virulence factors and the inhibition of biofilm formation by C. violaceum ATCC 12472 were investigated in the present study at sub-MICs without inhibiting planktonic growth. Our results show that ZnO-NPs with an average diameter of 40 nm reduce the production of violacein in C. violaceum ATCC 12472 due to the downregulation of *cviI* and *vioA*. The change in violacein production has been attributed to the disruption of processes related to signal perception by the QS system (31). In addition, the results show the significant inhibition of the formation of biofilms treated with ZnO-TBQ. We next determined the expression levels of biofilm-related genes. The expression of *hmsP* is prominently suppressed, which is well correlated with the results of the biofilm formation assay. This result is in agreement with the hypothesis that ZnO-TBQ showed a synergistic effect on the biofilm formation of C. violaceum ATCC 12472. These results thus reveal a sophisticated relationship between QS and biofilms. In addition, after ZnO-TBQ treatment, it was observed that biofilms were scattered and flat. The changed biofilm architecture and membrane permeability facilitate the penetration of TBQ into the treated biofilms, as evidenced by the dominant red fluorescence in the combination groups. We therefore speculated that the architecture of the preformed biofilms was destroyed. Meanwhile, the present results suggest that ZnO-TBQ, compared with ZnO-NPs or TBQ, shows higher-level activity in inhibiting virulence factors and biofilm formation. Hence, ZnO-TBQ showed a synergistic effect with QS-inhibitory effects on C. violaceum ATCC 12472.

These results indicated that both TBQ and ZnO-NPs inhibited virulence factors and biofilm formation. However, the mechanism of action of ZnO-NPs remains unclear. Three possible mechanisms have been reported for the antibacterial activity of ZnO-NPs in the absence of a light source, but few of them have referred to QSI activity. First, the generation of oxygen radicals or hydrogen peroxide (H_2_O_2_) produced from the surface of ZnO-NPs via oxygen defect sites could be responsible for the antibacterial activity, even in the dark (32, 33). However, the concentrations of ZnO-NPs that those researchers used in their experiments were 100 μg/mL and 500 μg/mL, respectively, which were higher than the concentration used in this work (25 μg/mL). Accordingly, ZnO-NPs displayed no significant inhibition of the proliferation of C. violaceum ATCC 12472 at 25 μg/mL (Fig. 1A) in our study, indicating that there is no generation of oxygen radicals or H_2_O_2_ or that the concentration is not high enough to inhibit the proliferation of C. violaceum ATCC 12472. Second, the dissolution of Zn^2+^ ions from ZnO-NPs is another proposed mechanism for the antimicrobial activity (24, 34). Nonetheless, it was determined that the concentration of released Zn^2+^ ions is not sufficient to induce bacterial toxicity (35). Third, another proposed mechanism is damage to the bacterial cell wall through the transfection of ZnO-NPs (36). However, the above-described studies covered mainly antimicrobial activity at higher concentrations (≥100 μg/mL) instead of QSI activity. To evaluate whether the effects on the virulence factors and biofilm of C. violaceum ATCC 12472 at lower concentrations arise from Zn^2+^ ions, ZnO-NPs, or other types of nanoparticles, we investigated the inhibitory effects of Zn(NO_3_)_2_ (1.6 μg/mL [the solubility of ZnO]), Zn(NO_3_)_2_ (1.6 μg/mL)-TBQ (25 μg/mL), ZnO-NPs, ZnO-TBQ, TiO_2_-NPs, and TiO_2_-TBQ (25 μg/mL each) on violacein, biofilms, and cell viability. ZnO-NPs had a greater inhibitory effect on violacein (see Fig. S1 in the supplemental material) and cell viability (Fig. S2B) than did Zn(NO_3_)_2_ alone and TiO_2_-NPs, which suggests that the concentration of dissolved Zn^2+^ ions in ZnO-dispersed medium was too low to reduce violacein and bacterial viability in biofilms (34). Compared with TiO_2_-TBQ, ZnO-TBQ possesses a conspicuous inhibitory effect on violacein and biofilm formation (Fig. S2A), indicating that the inhibitory effect depends on both TBQ and the characteristics of the nanoparticles rather than the nanoparticulate form as such. Although it is still unclear how ZnO-NPs attach to the bacterial cell wall and dissolve to release Zn^2+^ ions, lipoteichoic acid on the outer membrane and exopolysaccharide in biofilms of Gram-negative bacteria, because of their negative charge, might contribute to attracting the ZnO-NPs (37). Consequently, they may provide attachment sites for ZnO-NPs. Under normal conditions, bacterial metalloproteins strictly restrict metal occupancy based on the binding affinity of transition metals, known as the Irving-Williams series of metals (38). Under abnormal conditions and at high concentrations of Zn^2+^ ions, the influx transporters for noncompetitive metals might lose their critical selectivity and eventually transport Zn^2+^ ions instead of noncompetitive metal ions. According to our experimental data and previous literature reports, the following mechanism of action of ZnO nanoparticles can be hypothesized. After the addition of ZnO-NPs to the medium, they will attach to cell walls or biofilms, and the local dissolution of ZnO-NPs can lead to increased Zn^2+^ concentrations, which could destroy metal homeostasis, corresponding to disturbances in amino acid metabolism and nucleic acid metabolism. Although the local dissolution of the attached ZnO-NPs can lead to increased Zn^2+^ concentrations, the concentration of dissolved Zn^2+^ ions in ZnO-dispersed medium (1.6 μg/mL) was too low to reduce violacein and bacterial viability in biofilms. Instead of ZnO-NPs, TBQ could also be combined with Zn^2+^ ions, but a high concentration of Zn^2+^ ions would be required. However, Zn^2+^ ions possess cell toxicity at concentrations of >30 mg/L and may cause a pollution risk. Thus, ZnO nanoparticles present a more potent application under many conditions than Zn^2+^ ions.

C. violaceum ATCC 12472 uses *N*-decanoyl-homoserine lactone (C_10_-HSL) as a QS signal to mediate the expression of multiple genes involved in a variety of physiological activities, including virulence production and biofilm formation (39). The QS signal C_10_-HSL of C. violaceum ATCC 12472 is synthesized by *cviI* and then specifically binds to *cviIR* to induce the expression of a range of proteins involved in virulence factor production and biofilm maturation (40). Our results show the significant downregulation of the *cviI* and *cviR* genes after treatment with TBQ-ZnO. The *cviR* gene regulates the expression of *vioA*, *vioB*, and *vioC* after the activation of C_10_-HSL (41). The *vioA* gene, a flavoenzyme l-tryptophan oxidase gene that regulates the oxidative conversion of l-tryptophan to violacein (42), was downregulated by 86%, corresponding to the disturbances in the phenylalanine, tyrosine, and tryptophan biosynthesis metabolism pathway (Fig. 6A). The *chiA* gene, which is related to the synthesis of chitinase, was downregulated by 74%. Compared with TBQ, the inhibition rate of ZnO-TBQ increased by 47%. The *hmsP* gene, encoding a bifunctional diguanylate cyclase/phosphodiesterase that is related to biofilm formation (43), was downregulated by 87%, contributing to the disturbances in glycerophospholipid metabolism. The *lolA* gene was also downregulated by 85%. This gene encodes a periplasmic chaperone that enables lipoproteins to be transferred from the cells’ inner to outer membranes (44). All in all, ZnO-TBQ effectively reduces the expression of genes related to QS, which is conducive to limiting the infectivity of C. violaceum ATCC 12472 and leads to disturbances in amino acid metabolism, organic acid metabolism, and nucleic acid metabolism, among others. Furthermore, in an *in vivo* assay, ZnO-TBQ treatment of C. elegans nematodes presented a significant improvement in the survival rate by limiting the infectivity of C. violaceum ATCC 12472. Similarly, it has also been reported that linalool could be used as a QSI leading to high survival rates in C. elegans compared to the infection control (45).

The combination of a QS inhibitor and a conventional antibiotic is known as a promising approach for eradicating preformed biofilms and decreasing the magnitude of the infection (16). Alternatively, we suggest that the combination of TBQ and ZnO-NPs could reduce the use of antibiotics and, consequently, attenuate the risk of antibiotic resistance. In this study, we found that ZnO-TBQ showed a synergistic effect with QS-inhibitory effects on C. violaceum ATCC 12472.

## MATERIALS AND METHODS

### Chemicals and strains.

ZnO-NPs (<100-nm particle size [TEM], ≤40-nm average particle size [APS], 20 wt% in H_2_O) and other chemicals were purchased from Sigma-Aldrich (St. Louis, MO, USA). C. violaceum ATCC 12472 was received from the Guangdong Provincial Center for Microbial Strains (Guangzhou, China). The bacteria were cultured at 28°C in Luria-Bertani (LB) medium (Sangon Biotech Co., Ltd., Shanghai, China).

### Determination of MICs and growth curves.

The MICs of TBQ, ZnO-NPs, and ZnO-TBQ against C. violaceum ATCC 12472 were determined in a polystyrene 96-well plate (Corning, USA) according to the methods of the Clinical and Laboratory Standards Institute (CLSI) (46), with minor modifications. The strain was inoculated into 5 mL LB broth at 28°C at 170 rpm for 17 h and then adjusted to an optical density at 620 nm (OD_620_) of 0.05 with fresh LB broth, which corresponds to a resulting bacterial concentration of about 1.5 × 10^5^ to 2.0 × 10^5^ CFU/mL (47). Next, 190 μL of the diluted cultures was added to each well, and LB medium served as a blank. Next, 10 μL of a TBQ, ZnO-NP, or ZnO-TBQ (1:1, M/M) solution was added to each well at different final concentrations (12.5, 25, 50, 100, 125, 250, and 500 μg/mL, respectively). Afterward, the mixtures were cultured at 28°C at 170 rpm for 24 h. Dimethyl sulfoxide (DMSO) was used as a negative control. The absorbance was measured every 4 h at 620 nm (OD_620_) using a microplate reader (ELx800; BioTek, Winooski, VT, USA). Three replicates were performed for each experiment.

### Virulence factor assays.

**(i) Violacein.** Based on previously reported methods, with minor modifications (48), C. violaceum ATCC 12472 was inoculated into 5 mL LB broth at 28°C at 170 rpm for 17 h. Next, the cultures were inoculated into fresh LB broth (1:100, vol/vol) supplemented with TBQ, ZnO-NPs, or ZnO-TBQ (25 μg/mL each) (49). DMSO was used as a negative control. After incubation at 28°C at 170 rpm for 24 h, 1 mL of the culture solution was centrifuged at 10,000 rpm for 10 min. One milliliter of acidified ethanol (4%) (1 M HCl) was added to the bottom precipitated bacteria, and the solution was centrifuged again at 10,000 rpm for 5 min. The absorbance was then determined at 534 nm using the microplate reader.

**(ii) Hemolysin.** After centrifugation of the above-described bacterial liquid at 10,000 rpm for 10 min, the supernatant was mixed with a sheep blood suspension (1:9, vol/vol) and then incubated at 28°C for 1 h. After centrifugation at 3,000 rpm for 10 min, the OD_530_ was determined (50).

**(iii) Chitinase activity.** A micro chitinase assay kit (Beijing Solarbio Co., China) was used to test the chitinase activity, according to the manufacturer’s instructions. A total of 0.5 mL of the culture material was carefully mixed with the standard solution, centrifuged at 10,000 rpm for 20 min, treated in a boiling water bath for 10 min, and immediately placed on ice. The absorbance was measured at 540 nm (51).

### Biofilm formation assays.

To evaluate whether TBQ, ZnO-NPs, and ZnO-TBQ can inhibit biofilm formation, C. violaceum ATCC 12472 cultures grown overnight were inoculated into fresh LB broth (1:100, vol/vol) supplemented with TBQ, ZnO-NPs, ZnO-TBQ (25 μg/mL each), or DMSO (negative control) (52). After incubation at 28°C for 24 h, the biofilms were gently rinsed with phosphate-buffered saline (PBS) (pH 7.2) three times to remove planktonic cells and fixed with 200 μL methanol for 15 min, and the methanol was removed. After the plate was dried at 60°C, 100 μL of 0.05% crystal violet dye in ethanol was added to each well, and the mixture was incubated at room temperature for 15 min. The wells were washed three times with PBS (pH 7.2) to remove the nonattached dye and then dried again at 60°C. One hundred fifty microliters of 95% ethanol was added to each well and decolorized at room temperature with shaking at 170 rpm for 15 min. The OD_570_ of the decolorization solution was determined using a microplate reader.

To quantify biofilm cell viability, the above-described biofilms were gently rinsed three times with PBS (pH 7.2). The exopolysaccharides of the above-described biofilms were then digested with 5 U glucanase (catalog number D8144; Sigma-Aldrich, St. Louis, MO, USA) at 37°C for 30 min (53) and then sonicated for 30 s at 40 Hz (catalog number KQ-250; Kunshan Ultrasound Instrument Co., Ltd., Suzhou, China). The number of CFU per biofilm was quantified by a spreading plate assay with LB agar medium (Sangon Biotech, Shanghai, China), with incubation at 28°C overnight.

### Scanning electron microscopy.

Scanning electron microscopy (SEM) measurements were carried out according to methods described previously (54), with slight modifications. SEM was used to observe the shape of the biofilms on glass substrates after treatment with TBQ, ZnO-NPs, ZnO-TBQ (25 μg/mL each), or DMSO (negative control) in 24-well polystyrene plates (Costar 3524; Corning Inc., Corning, NY, USA). After incubation at 28°C for 24 h, the biofilms were rinsed with sterile PBS (pH 7.2), fixed with 4% glutaraldehyde (obtained by dilution with 0.1 M PBS, pH 7.2) at 4°C for 4 h, and dehydrated by gradient elution with different ethanol-PBS mixtures of 50, 70, 80, 90, and 100% in turn. After freeze-drying, the samples were coated with gold and taken for SEM measurements (JSM6360; JEOL, Tokyo, Japan).

### Confocal laser scanning microscopy.

Confocal laser scanning microscopy (CLSM) measurements were performed with a Zeiss LSM 700 microscope (Carl Zeiss, Jena, Germany) as reported previously (13), with minor modifications. The culture process was the same as the one described above for SEM samples. After incubation at 28°C for 24 h, the biofilms that formed on the coverslips were rinsed three times with sterile PBS (pH 7.2). Next, the biofilms were stained with a 0.01% acridine orange–ethidium bromide solution for 15 min and taken for CLSM measurements. Images were collected at 485-nm excitation and 535-nm emission wavelengths.

### RT-qPCR.

Real-time quantitative PCR (RT-qPCR) was performed according to a previously reported method, with modifications (48). The cultures treated with TBQ, ZnO-NPs, ZnO-TBQ (25 μg/mL each), or DMSO (negative control) were incubated at 28°C for 24 h. Next, the cell pellets were collected from 1 mL of the bacterial samples at 12,000 rpm for 10 min. Total RNA was extracted using a bacterial total RNA extraction kit (Tianjin Biotechnology Co., Ltd., Beijing, China) according to the manufacturer’s instructions. Reverse transcription amplification was performed with Golden star RT6 cDNA synthesis kit version 2 of the Jinke Gold reverse transcription kit. Oligonucleotide primers for RT-qPCR are shown in Table S2 in the supplemental material. RT-qPCR was performed using a LightCycler 96 instrument (Roche Diagnostics, USA) with MonAmp SYBR green qPCR mix (Monad, China) according to the manufacturers’ recommendations. All measurements were performed in triplicate, and the housekeeping gene *rpsL* was used as an internal reference for normalization. Relative fold changes in gene expression were calculated using the 2^−ΔΔ^*^CT^* method.

10.1128/msphere.00597-22.8 TABLE S2 PCR primers for RT-qPCR. Download Table S2, PDF file, 0.1 MB. Copyright © 2023 Liu et al. 2023 Liu et al. https://creativecommons.org/licenses/by/4.0/ This content is distributed under the terms of the Creative Commons Attribution 4.0 International license.

### Molecular docking analysis.

The initial dimensional geometric coordinates of the X-ray crystal structure of CviR were downloaded from the Protein Data Bank (PDB) (https://www.rcsb.org/) under accession number 3QP8. The centroids of the native ligand in the crystal structure were defined as the centers of the binding pockets in the docking process. Docking modes were generated by AutoDock4. According to the docking protocol (55), all water molecules were deleted in the CviR complex. We employed the AutoDock protocol as a docking approach to carry out semiflexible docking. We set other parameters to default values (56).

### LC-MS-based analysis of bacterial metabolites.

Cultures of C. violaceum ATCC 12472 incubated at 28°C overnight were added to LB broth (1:100, vol/vol) containing TBQ, ZnO-NPs, ZnO-TBQ (25 μg/mL each), or DMSO (negative control) and then incubated at 28°C for 24 h. Subsequently, the bacteria were separated by centrifugation at 10,000 rpm at 4°C for 10 min, and the precipitate was washed with PBS. Afterward, the metabolites were homogenized. The freeze-dried bacterial metabolites were dissolved in methanol and analyzed by UHPLC-QE-MS. The metabolites were assigned according to the KEGG library. All statistical analyses of liquid chromatography-mass spectrometry (LC-MS) data and graphics were performed in triplicate by using RStudio (R version 3.5.2) (57).

### C. elegans survival assay.

A C. elegans survival assay was performed according to methods reported in the literature, with slight modifications (58, 59). Briefly, 20 μL of a broth culture of C. violaceum ATCC 12472 grown overnight was added to a 6-cm nematode growth medium (NGM) plate containing 70 μM 5′-fluoro-2′-deoxyuridine and incubated at 28°C for 24 h to create a bacterial lawn. Next, 30 synchronized nematodes (L4 stage) of the C. elegans N2 strain were selected, suspended on the bacterial lawn, and incubated at 25°C for 12 h. After incubation, the infected nematodes were washed with M9 buffer, and the rinsed nematodes were transferred to each well of a 24-well plate containing 10% LB medium in M9 buffer with TBQ, ZnO-NPs, ZnO-TBQ (each at 25 μg/mL), and DMSO. The plates were incubated at 25°C, and nematodes were counted every 24 h using a stereomicroscope. The dead nematodes were removed, and the living ones were transferred to a new plate until all nematodes were dead. The number of nematodes that survived was tabulated to generate a survival curve.

### Statistical analysis.

Analysis of variance (ANOVA) was performed by comparing the differences between groups using SPSS statistics software (version 18.0; SPSS Inc., Chicago, IL). All experiments were repeated three times, followed by Tukey-Kramer tests. Differences were considered statistically significant when the *P* values were ≤0.05.
