# Supplementary material for: Combination of 2-tert-Butyl-1,4-Benzoquinone (TBQ) and ZnO Nanoparticles, a New Strategy To Inhibit Biofilm Formation and Virulence Factors of Chromobacterium violaceum
Source: mSphere. 2023 Jan 16;8(1):e00597-22. doi: 10.1128/msphere.00597-22 (PMC9942565; doi:10.1128/msphere.00597-22)
Supplement: TABLE S1 [file msphere.00597-22-s0007.pdf]

Table S1 MIC values of ZnO-NPs, TBQ, and ZnO-TBQ.

Agent	MIC (μg/mL)
	C. violaceum ATCC12472
TBQ	100
ZnO-NPs	100
ZnO-TBQ	50
