# Supplementary figures and images for: Combination of 2-tert-Butyl-1,4-Benzoquinone (TBQ) and ZnO Nanoparticles, a New Strategy To Inhibit Biofilm Formation and Virulence Factors of Chromobacterium violaceum
Source: mSphere. 2023 Jan 16;8(1):e00597-22. doi: 10.1128/msphere.00597-22 (PMC9942565; doi:10.1128/msphere.00597-22)

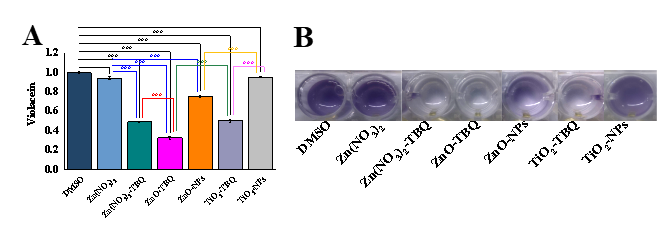

Supplement: FIG S1 [file msphere.00597-22-s0001.tif]

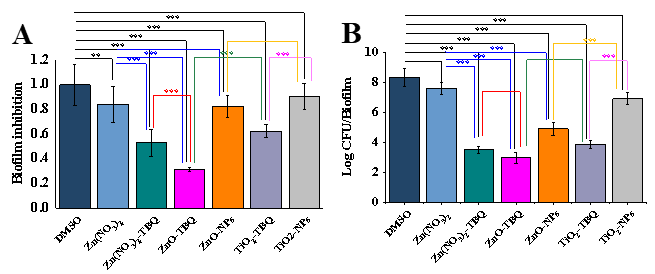

Supplement: FIG S2 [file msphere.00597-22-s0002.tif]

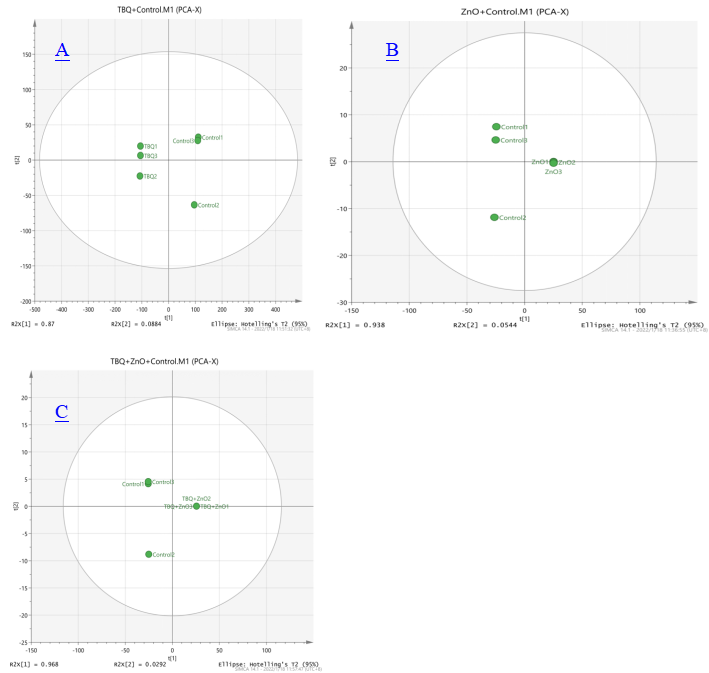

Supplement: FIG S3 [file msphere.00597-22-s0003.tif]

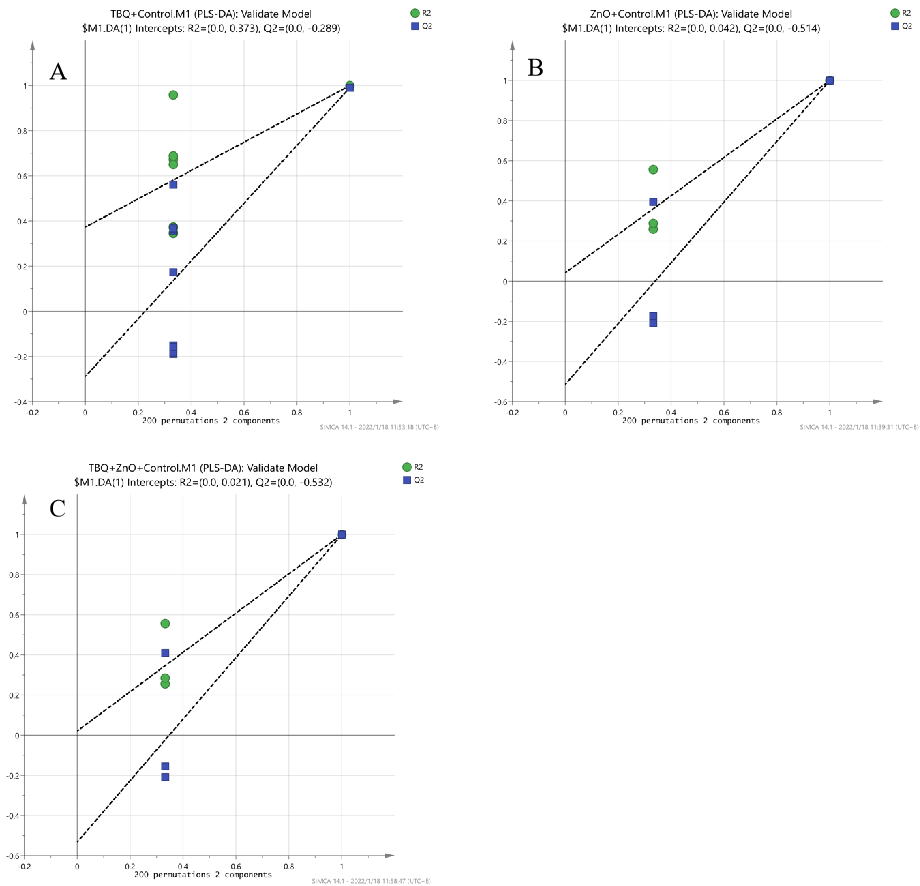

Supplement: FIG S4 [file msphere.00597-22-s0004.tif]

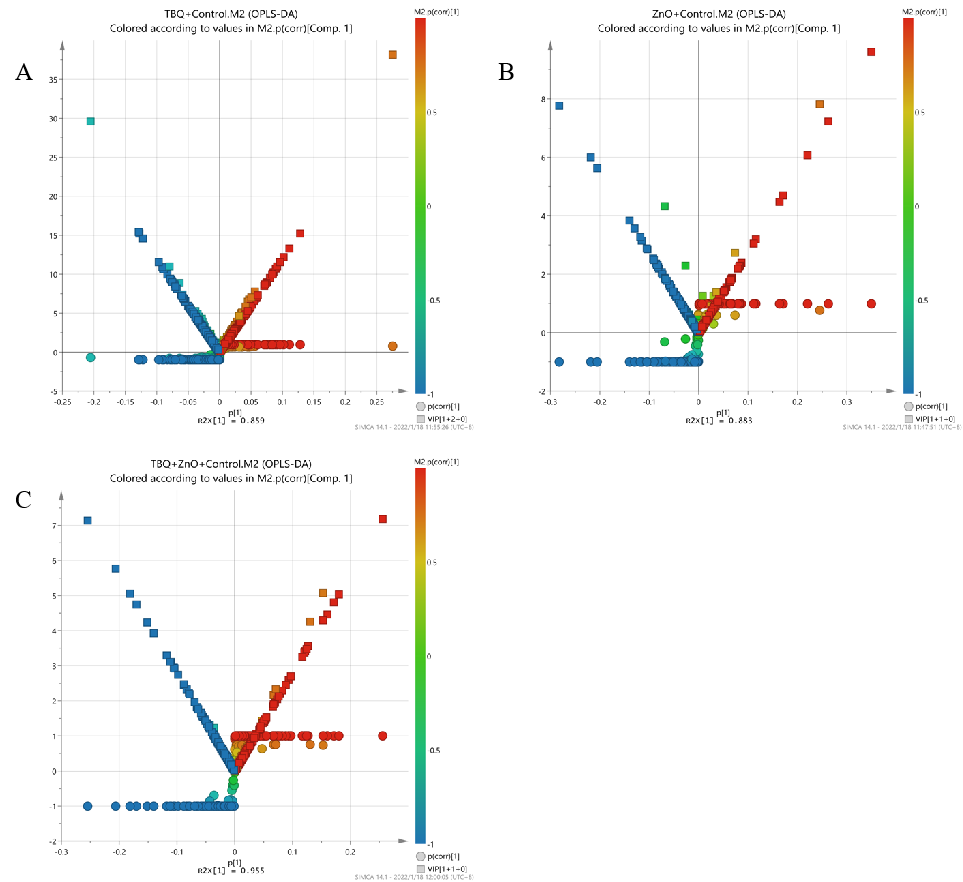

Supplement: FIG S5 [file msphere.00597-22-s0005.tif]

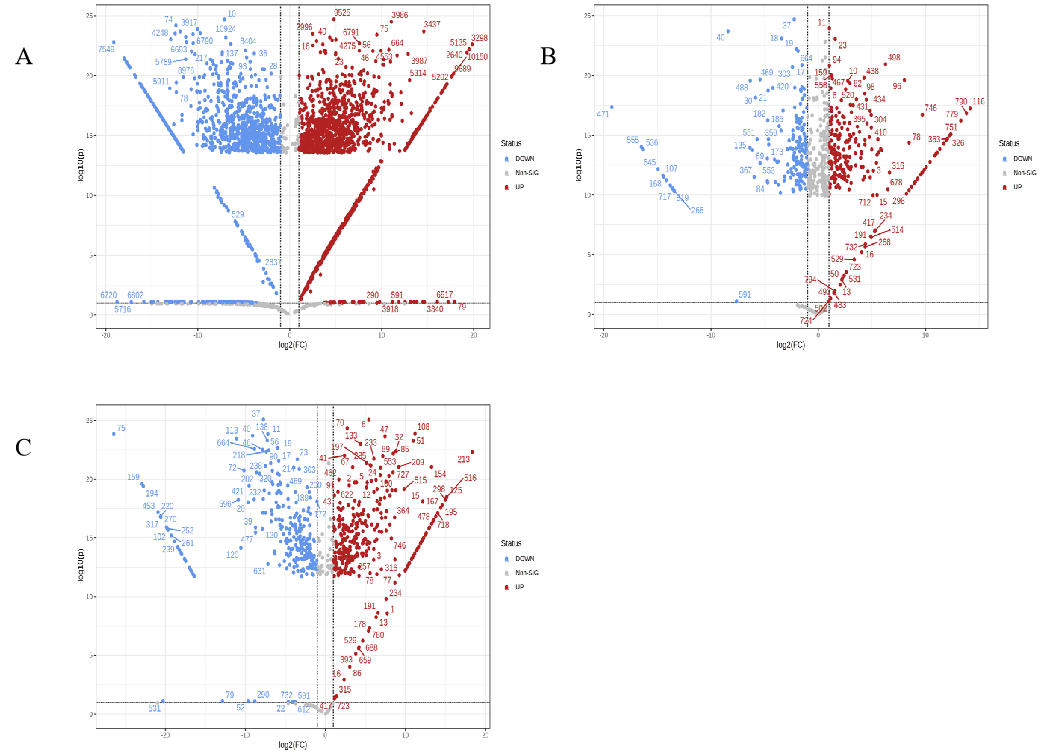

Supplement: FIG S6 [file msphere.00597-22-s0006.tif]
