# Supplementary material for: Combination of 2-tert-Butyl-1,4-Benzoquinone (TBQ) and ZnO Nanoparticles, a New Strategy To Inhibit Biofilm Formation and Virulence Factors of Chromobacterium violaceum
Source: mSphere. 2023 Jan 16;8(1):e00597-22. doi: 10.1128/msphere.00597-22 (PMC9942565; doi:10.1128/msphere.00597-22)
Supplement: TABLE S2 [file msphere.00597-22-s0008.pdf]

Table S2 PCR primers for RT-qPCR

Gene	Function	Primer direction	Sequence (5'-3')
cviI	LuxI family AHL synthase	Forward	TATCCAGCACAGGCACATCA
		Reverse	GGATGCGTGGTCATAGAAGG
cviR	LuxR family transcriptional regulator	Forward	ATGGGATGTCGCAATAGAGG
		Reverse	GTCCTGATGGCGCAAGAA
chiA	Chitinase	Forward	CGGCCGTAGTATTTCTTCCA
		Reverse	TTACAACACCGAGTGCAACG
hmsP	Bioflim formation regulator	Forward	CTAGAGCAAGCGTTGCTGAAAC
		Reverse	ACCGAGGCGCAGCTGG
lolA	Outer membrane lipoprotein chaperone	Forward	ACCAGACCGTGACCAACAAG
		Reverse	TGTAGCTGCGCTCGATTTCA
vioA	Tryptophan-2-monooxygenase	Forward	GCTGTTCAAGGCTTTCCTCA
		Reverse	GGTTGTCGACGATCAGCAC
rpsL	Internal reference	Forward	GCAACTATCAACCAGCTGGTG
		Reverse	GCTGTGCTCTTGCAGGTTGTG
